# Self-care strategies that support PrEP adherence: a qualitative study with female sex workers in rural Uganda

**DOI:** 10.1080/26410397.2025.2560175

**Published:** 2025-09-12

**Authors:** Lydia Jacenta Nakiganda, David Serwadda, Rosette Nakubulwa, Benjamin R. Bavinton, Andrew E. Grulich, Stephen Bell

**Affiliations:** a Research Fellow, Rakai Health Sciences Program, Entebbe, Uganda.; b Principal Investigator, Rakai Health Sciences Program, Entebbe, Uganda; Professor, Makerere University School of Public Health, Kampala, Uganda; c Research Assistant, Rakai Health Sciences Program, Entebbe, Uganda; d Associate Professor, Kirby Institute for Infection and Immunity in Society, UNSW Sydney, Sydney, Australia; e Professor, Kirby Institute for Infection and Immunity in Society, UNSW Sydney, Sydney, Australia; f Professor, Burnet Institute, Melbourne, Australia

**Keywords:** self-care, HIV prevention, female sex workers, Uganda, PrEP, qualitative, people-centred, health systems

## Abstract

The World Health Organization (WHO) recently released revised guidelines on self-care interventions for health and well-being, which, in 2022, included recommendations supporting equitable access to information about and availability of pre-exposure prophylaxis (PrEP) as a self-care strategy. Successful implementation of PrEP as an HIV prevention strategy extends beyond providing access to medication. It hinges on individuals adopting self-care strategies to ensure adherence to PrEP in their daily lives. This paper aims to explore self-care strategies that bolster adherence to oral PrEP among female sex workers in two rural Ugandan settings. Through in-depth interviews with 20 female sex workers residing in fishing communities or Trans-Africa highway towns, we used deductive thematic analysis to explore people-centred and health system-centred perspectives on women’s PrEP-related self-care strategies. A people-centred perspective on self-care illustrated a range of self-care strategies to support PrEP adherence conducted by women individually (medication reminders; pairing PrEP with daily habits), and with support from others in familial and social networks (verbal reminders to take tablets; information sharing; shared clinic visits; shared pill-taking routines). A health system-centred perspective illustrated the importance of support from health services and professionals. Examples included information provision; NGO clinics as friendly, safe, non-judgemental spaces; PrEP distribution through home-based care outreach strategies; in-bulk PrEP provision for work-related travel periods; formal integration of female sex workers into the system as peer health workers. By considering both person-centred and health system-centred perspectives on self-care, we can pinpoint strategies for health systems to assist female sex workers and their communities in preventing the acquisition and transmission of HIV.

## Plain language summary

The World Health Organization (WHO) recently updated its guidelines to support the use of pre-exposure prophylaxis (PrEP) as a self-care strategy for HIV prevention. Effective PrEP use relies on individuals incorporating it into their daily lives, with or without support from health systems. This paper examines the self-care strategies that help female sex workers in rural Uganda adhere to PrEP. Through interviews with 20 women, we identified people-centred and health system-centred support for self-care strategies that enabled PrEP adherence. By understanding people-centred and health system-centred perspectives on self-care to support PrEP, we can better support these women and their communities in preventing HIV.

## Résumé

L’Organisation mondiale de la Santé (OMS) a récemment révisé ses lignes directrices sur les interventions d’auto-prise en charge pour la santé et le bien-être qui, en 2022, incluaient des recommandations en faveur d’un accès équitable aux informations sur la prophylaxie préexposition (PrEP) et sa disponibilité comme stratégie d’auto-prise en charge. La mise en œuvre réussie de la PrEP comme stratégie de prévention du VIH va au-delà de l’accès aux médicaments. Elle repose sur l’adoption par les individus de stratégies autogérées pour garantir l’observance de la PrEP dans leur vie quotidienne. Cet article vise à étudier les stratégies d’auto-prise en charge qui stimulent l’observance de la prophylaxie orale chez les travailleuses du sexe dans deux zones rurales ougandaises. Au moyen d’entretiens approfondis avec 20 travailleuses du sexe résidant dans des communautés de pêcheurs ou des villes situées le long de la route transafricaine, nous avons utilisé une analyse thématique déductive pour étudier les perspectives centrées sur les personnes et sur le système de santé concernant les stratégies d’auto-prise en charge des femmes pour la PrEP. Une perspective centrée sur la personne en matière d’auto-prise en charge a illustré une série de stratégies autogérées visant à faciliter l’observance de la PrEP mises en œuvre par les femmes individuellement (rappels des médicaments; association de la PrEP avec les habitudes quotidiennes), et avec le soutien d’autres membres de leur famille ou des réseaux sociaux (rappels oraux de prendre les comprimés; partage d’informations; visites communes aux centres de santé; routines partagées de prise des pilules). Une perspective centrée sur le système de santé a illustré l’importance du soutien des services et des professionnels de santé. Parmi les exemples, on peut citer la diffusion d’informations; les centres d’ONG comme espaces conviviaux, sûrs et sans jugement; la distribution de la PrEP par le biais de stratégies de soins à domicile; la fourniture de PrEP en gros pour les périodes de déplacement professionnel; l’intégration formelle des travailleuses du sexe dans le système en tant qu’agents de santé pairs. En envisageant à la fois les perspectives centrées sur la personne et celles centrées sur le système de santé en matière d’auto-prise en charge, nous pouvons mettre en évidence des stratégies qui permettent aux systèmes de santé d’aider les travailleuses du sexe et leurs communautés à prévenir la contamination et la transmission du VIH.

## Resumen

La Organización Mundial de la Salud (OMS) recientemente publicó directrices modificadas sobre las intervenciones de autocuidado para la salud y el bienestar, que, en 2022, incluían recomendaciones a favor del acceso equitativo a la información sobre y la disponibilidad de la profilaxis preexposición (PrEP) como una estrategia de autocuidado. La aplicación exitosa de la PrEP como una estrategia de prevención del VIH se extiende más allá de ofrecer acceso al medicamento. Depende de que las personas adopten estrategias de autocuidado para garantizar adherencia a la PrEP en su diario vivir. Este artículo pretende explorar las estrategias de autocuidado que refuerzan la adherencia a la PrEP oral entre trabajadoras sexuales en dos entornos rurales de Uganda. Por medio de entrevistas a profundidad con 20 trabajadoras sexuales que residían en comunidades pesqueras o pueblos a lo largo de la Carretera Transafricana, utilizamos el análisis temático deductivo para explorar perspectivas centradas en la persona y centradas en el sistema de salud sobre las estrategias de autocuidado de las mujeres relacionadas con la PrEP. La perspectiva centrada en la persona sobre el autocuidado ilustró una variedad de estrategias de autocuidado para apoyar la adherencia a la PrEP realizada por cada mujer (recordatorios del medicamento; combinar la PrEP con hábitos diarios), y con el apoyo de otras personas en redes familiares y sociales (recordatorios orales de tomarse las tabletas; intercambio de información, visitas compartidas a los centros de salud; rutinas compartidas de toma de pastillas). La perspectiva centrada en el sistema de salud ilustró la importancia de que las mujeres cuenten con el apoyo de los servicios y profesionales de salud, por ejemplo: provisión de información; las clínicas de ONG son espacios amigables, seguros y libres de prejuicios; la distribución de PrEP por medio de estrategias de extensión de atención domiciliaria; provisión de PrEP en grandes cantidades para períodos de viaje relacionado con el trabajo; integración formal de las trabajadoras sexuales en el sistema como promotoras de salud. Al considerar tanto las perspectivas de autocuidado centradas en la persona como aquellas centradas en el sistema de salud, podemos identificar las estrategias que los sistemas de salud pueden utilizar para ayudar a las trabajadoras sexuales y sus comunidades a prevenir la adquisición y transmisión del VIH.

## Introduction

In 2022, the World Health Organization (WHO) published revised guidelines on self-care interventions for health and well-being.^1^ These guidelines defined self-care as, “the ability of individuals, families and communities to promote health, prevent disease, maintain health and cope with illness and disability with or without the support of a health worker”, and self-care interventions as including “evidence-based, high-quality drugs, devices, diagnostics and/or digital interventions that can be provided fully or partially outside formal health services and be used with or without a health worker”.^1^ Self-care is not a new concept; people have been practicing these types of activities for millennia across diverse social and cultural settings.^2^ However, in an international context which includes the push for universal health coverage, people-centred approaches to healthcare, overstretched health systems and global shortages of trained health workers, self-care has been revitalised as an important component in efforts to achieve health and well-being for populations around the world.

Self-care approaches are perceived as particularly powerful in the context of sexual rights, enabling marginalised and oppressed people greater autonomy and agency in health seeking and care strategies in contexts where access to formal healthcare is heavily stigmatised.^1,3–5^ In 2019, Narasimhan et al published a new framework for self-care based around two perspectives: a people-centred framing, which focussed on improving the capacity of individuals to make informed decisions and make use of available health, societal and familial resources; and a system-centred framing, focussed on how self-care relates to health systems through, for example, task shifting from health professionals to the public, or optimised linkage to care.^3^ The framework proposes interrelated domains of self-care, including: self-carers and care givers; their self-care activities and strategies; the places where self-care occurs; and a safe and supportive enabling environment for self-care.^3^ While individuals can be in control of some self-care activities (e.g. using condoms), other self-care activities require interaction with the health system (e.g. confirmation of a positive HIV test result after self-testing).^3^

HIV self-testing was a priority in WHO’s earlier self-care guidelines in 2019.^6^ In 2022, guidelines included a focus on access to pre-exposure prophylaxis (PrEP) – the use of antiretroviral medication to prevent the acquisition of HIV – with recommendations supporting the offer of, equitable access to, information about and availability of PrEP as a self-care strategy.^1^ PrEP is recommended for populations at risk of HIV infection internationally^7^ and in Uganda^8^ including for female sex workers and their clients. Studies in Ugandan^9–11^ and other sub-Saharan settings^12–14^ indicate high levels of acceptability of PrEP among sex workers. But effectiveness of PrEP as a strategy to limit the acquisition and transmission of HIV is dependent on people feeling able to access PrEP from supportive, safe services,^15,16^ and health facilities having the resources and capacity to support PrEP adherence.^17,18^ Beyond the provision of, access to and availability of medicine, the success of PrEP as an HIV prevention strategy is also dependent on people adopting self-care strategies that enable PrEP adherence in their daily lives.

This paper aims to explore self-care strategies that support adherence to oral PrEP among female sex workers living and working in two rural Ugandan settings. By drawing on narratives collected from 20 female sex workers, we contribute to the current conceptualisation of self-care by exploring both people-centred and system-centred perspectives on PrEP adherence, and the dynamic interaction between individuals and health systems. We also highlight activities and initiatives in community and health service settings that can be implemented more systematically by government and NGOs in Uganda – and in settings beyond Uganda – to support HIV prevention by enabling PrEP as a self-care strategy among marginalised people, in spaces they deem safe to use.

## Methods

### Author reflexivity statement

Our research team comprised three Ugandan and three international researchers. LJN (PhD) is a Ugandan woman from Kyotera/Rakai, who has 10 years’ experience working as a researcher in Uganda, with a focus on HIV key populations. DS (PhD) – a Ugandan epidemiologist and physician, a professor and former Dean of the School of Public Health at Makerere University College of Health Sciences in Kampala, Uganda – has over 30 years’ clinical and community research experience in HIV and related fields. RN is a Ugandan woman with eight years’ experience in social-behavioural research. BRB (PhD), an Australian man with 20 years’ experience in HIV prevention and research, has led epidemiological and socio-behavioural research with gay, bisexual and other men who have sex with men, trans and gender diverse people, and other key populations in Australia and several low-to-middle income countries. AEG (PhD) – a medical epidemiologist, professor and specialist public health physician – has 30 years’ experience leading research on the prevention of HIV and sexually transmitted infections in gay, bisexual and other men who have sex with men conducting HIV research. SB (PhD) is a British man who holds Australian citizenship, has over 20 years’ experience in participatory community-based research in diverse international settings, and has led and supported HIV research with young people and key populations in Uganda since 2003. This study was a small component of LJN’s doctoral research; DS, BRB, AEG and SB were members of LJN’s supervision team. We recognise that our individual experiences and ways of relating to the world, each other and our work and research are diverse. Combined, we are a multidisciplinary team of researchers concerned with the health and well-being of people with HIV, and supportive of efforts to centre the knowledge and expertise of female sex workers in conceiving a future where they are able to live the lives they desire.

### Study design and setting

This paper is based on data collected during an exploratory qualitative study with 20 female sex workers living in Southwestern Uganda, approximately 200 km from the capital city, Kampala (Table 1). The study was guided by an interpretive approach that acknowledges the significance of accessing locally situated, subjective understandings of social life and social phenomena such as HIV, PrEP and care.^19,20^ Accessing these understandings contributed to the study aims by examining female sex workers’ experiences and social interactions with others that influence adherence to PrEP in rural settings in Uganda. The description of this methods section was guided by the COREQ checklist (see supplementary information).

**Table 1.** Participant characteristics

Participant characteristics	No. participants (% participants)
Age	
19–24 years	5 (25%)
25–30 years	9 (45%)
30–34 years	3 (15%)
35 + years	3 (15%)
Residency	
Fishing community	11 (55%)
Highway community	9 (45%)
Relationships status	
Living with long-term partner	4 (20%)
Single	16 (80%)
With children	
Yes	18 (90%)
No	2 (10%)
PrEP status	
Currently adhering To PrEP	12 (60%)
Stopped adhering to PrEP in the prior 3 months	8 (40%)

Data collection occurred between 7 June and 17 September 2021 in one town on the Trans-African Highway and one fishing community on the shores of Lake Victoria. The study locations are not named to protect the identity of study participants, but were identified as “HIV hotspots” by the Ugandan government.^21^ Hotspots are typically defined as spatial clusters of elevated disease burden or transmission risk and identified as improving the ability to reach populations at highest risk of HIV transmission.^22^

### Participants, sampling and recruitment

Study participants were sampled purposively^19^ to generate a broad understanding of the different lived experiences of PrEP use among female sex workers in the study settings. A form of non-probability sampling, purposive sampling occurs when researchers strategically select participants based on certain characteristics and experiences that are of relevance to the research question being investigated.^19^ For the purpose of this study, women were eligible to participate in the study if they self-identified as female; were aged 18 years or older; reported at least one instance of sex with a man in the preceding one month, involving the exchange of sex for money or gifts; resided in the study locations; and were either on PrEP for more than three months or had stopped taking PrEP less than three months before the interview. We aimed for equal numbers of participants from fishing and highway settings.

The target sample size was 20 participants. This number was chosen pragmatically to ensure diversity across the two study settings (fishing and highway) while remaining feasible given the study resources and timeframe. Although we did not explicitly plan recruitment around data saturation, we reached a point where no new major themes were emerging from the interviews, suggesting that thematic saturation was achieved.

Sex work is criminalised in Uganda,^23^ so snowball sampling techniques^19^ were used to identify and recruit participants safely and confidentially into the study. First, 20 women were recruited with assistance from “peer leaders”, defined as female sex workers who were trained by non-governmental organisations (NGOs) to support other sex workers with healthy living.^24^ Five peer leaders supported recruitment. Peer leaders identified women eligible to participate in the study from within their peer networks, introduced the study to them, and shared the lead author’s (LJN) contact details with those who were interested in participating in interviews. LJN held one-on-one meetings with each prospective participant at the participant’s preferred location to discuss study participation, anticipated risks and study purpose. Prospective participants were asked to consider their participation in the study and report back within the next three days, after which verbal consent was audio-recorded and data collection occurred. Second, interviewees referred other potential participants from within their peer networks to the lead author. Eight women were recruited using this form of snowball sampling; the same recruitment procedures were followed with these participants. Recruitment was completed once the target sample size was reached.

Interview transcripts were not returned to participants for review, primarily to protect their confidentiality and minimise risks associated with re-contacting women engaged in criminalised sex work. Instead, steps were taken during data collection to ensure participants’ perspectives were accurately captured, including iterative probing during interviews and careful attention to participants’ own wording and meanings.

### Data collection

A semi-structured interview guide – designed and piloted with peer leaders a month prior to data collection (data from these interviews were not included) – was used to explore participants’ experiences of PrEP. The interview guide was structured around several themes: experiences of being on PrEP; accessing PrEP from health services; PrEP use and disclosure within sexual relationships; and factors influencing PrEP adherence. Interviews lasted between 67 and 126 minutes and were conducted by LJN (in English) and RN (in Luganda and English), in audio-private locations chosen by participants. All interviews were audio-recorded, transcribed verbatim, translated from Luganda to English where relevant, and deidentified.

### Analysis

Interview data were uploaded into NVivo (version 12), a qualitative data management tool, in preparation for thematic analysis following Strauss and Corbin’s^25^ system of “open” and “axial” coding. Open coding involves reading through the narrative data to increase familiarity with the material and to prepare “theoretical memos”^25^ as analytical reminders for generating ideas and making links between different findings. Axial coding describes the later process of linking or organising open codes into themes and sub-themes, and providing evidence to support thematic findings.^25^ Drawing on Narasimhan et al's framework for self-care,^3^ we used deductive analytical techniques to code data into people-centred and health system-centred perspectives on self-care. Initial coding was conducted by LJN, and then reviewed by and discussed with RN and SB ensure consistency and understanding in line with the context and conceptual ideas related to self-care.

### Ethical considerations

Ethical approval was obtained from the Research and Ethics Committee of The AIDS Support Organization (TASOREC/036/2020-UG-REC-009, 21 May 2020), the Uganda National Council for Science and Technology (HS813ES, 1 March 2021) and UNSW Sydney’s Human Research Ethics Committee (HC200357, 29 May 2020). As approved by ethics committees, verbal consent was sought rather than written consent to protect participant identities and overcome fear of documentation due to the stigmatisation of sex work in Uganda. Pseudonyms are used throughout this paper to protect women’s identities.

## Findings

In total, 20 participants were involved in this study. Table 1 details participant characteristics.

### People-centred perspectives

#### Individual self-care strategies

Participants discussed a range of individual strategies that supported their use of PrEP for HIV prevention. Given the unpredictable, busy nature of their work, comprising long hours away from home and extreme tiredness, women adopted self-care strategies that enabled taking oral PrEP to become routine and convenient in their everyday lives. For instance, Maria (fishing community) set her phone alarm “to ring every day, every morning … and I take my pill”. She explained that this enabled her to establish a consistent habit:

“*It’s good to swallow one tablet at a specific time every day. It helps you not to forget … if you know the specific time or period when you swallow your pill, you can’t forget … when it’s the time you feel like something is missing and usually the phone alarm will remind you.*”

Other women integrated PrEP into their daily routines. Yozefa (fishing community) described using a “sex handbag” or “business handbag”, in which she carried “condoms, lubricants, money and some makeup”. She kept her PrEP bottles in the same bag, so that when she checked her “bag to make sure I have all things … usually my eyes go straight to the bottle [pills] … I then remember I must swallow my pills.” Paula (fishing community) drank water as soon as she woke up each day because “after some tiresome nights, water refreshens me, gives me energy for the day”, and takes her pill at the same time. Lucia (highway community) linked PrEP with prayer, saying, “I pray every time I wake up … As I pray sometimes, I remember I have to keep safe for my children. This always reminds me of a daily pill. I keep my pill bottle near my bed”. Jackie (fishing community) linked PrEP with her cosmetics, saying,

“*It is my lotion that reminds me of taking PrEP. My pill bottle is just next to my lotions, Vaseline and all my cosmetics. As I dress up in the mornings, the bottle is just there, my eyes go straight to the bottle, and I remember, ‘oh, pills’*.”

#### Interpersonal self-care strategies

Women talked about the importance of interpersonal support for PrEP adherence from long-term sexual partners, friends and neighbours. Several women explained that their sexual partners – who did not have HIV and were aware of their sex work – supported them to take pills every day, in part to avoid HIV entering their relationship. Gloria (highway community) described how her partner, whom she lives with, “will ask, ‘Did you take your drugs?’ … almost every day” because “he doesn’t want to get [HIV]”.

Women in serodiscordant relationships with HIV-positive men talked about shared pill-taking routines. This occurred particularly in fishing communities where NGOs had delivered HIV prevention activities for many years, where HIV testing was accessible, free and less stigmatised, and where many women working as sex workers tended to have an established relationship with a partner at home, and more regular long-term clients. Yozefa (fishing community) explained that her partner “doesn’t like condoms, so we go live [unprotected sex]” but that he was “very strict and does not want to infect me” so “reminds me every time to take my pills at night”. Jane (fishing community) explained that taking tablets with her partner had become a daily routine. She said they “take our pills at the same time at night”, whereby her long-time partner who is HIV-positive takes his “ARV [antiretroviral therapy] pills and I swallow my PrEP pills”.

In fishing and highway communities, similar interpersonal strategies to support PrEP adherence were established with neighbours and friends, some of whom were HIV negative and on PrEP, while others were HIV positive and taking ARVs. Yozefa (fishing community) explained that her neighbour is on ARVs, and “when it is 9 pm, we either call each other or knock at each one’s door to remind each other to take the medicine”. Nakidde (highway community) said she and her friend used “phones to call each other and remind ourselves about the time of taking the medicine”.

Friends could also be relied upon to support regular attendance at health clinics for refills each month. Winnie (fishing community) said that she and her friend, who was also a sex worker, “use the same boda-boda [motorcycle transport]” to “go to the same clinic for monthly refills”. She said her friend “goes for her ARVs, and I go for my PrEP”, and her friend “will call me to remind me of our clinic day for refills so that we go together”.

### Health system-centred perspectives

Women noted the importance of self-care strategies that were enabled by health services. For instance, women described how PrEP refill cards reminded them of their refill dates, the time and location of their appointment, and in some instances, the health worker in charge of health units and responsible for the provision of refills. Nakidde (highway town) explained that she was given a card when she started taking PrEP to “show you when you are to visit the clinic for your medicine. Every time you come back, they fill in the next date and show you.” Florence (fishing community) said, “I can’t forget. I have that card. I know that on such a date, I am supposed to go back for a refill”.

Despite sex work being illegal and working conditions difficult, in fishing communities, PrEP adherence was also centred in trusted relationships with health workers that were forged over time, and embedded within social interactions that Jackie (fishing community) described as “friendly and professional”. Such relationships established between women and health workers were sometimes supported by outreach work for home-based delivery of PrEP. Immy (fishing community) explained that she “was able to keep my refill dates because of my relationship with *Musawo* [health worker]”. She described her home as being far away from the health service, but, when she had only several tablets left, “even when I could not come to the facility due to some reasons, I could call the health worker or ask the health worker to bring the medicine to my home”.

For other women, “peer health workers” supported PrEP adherence and were trusted sources of informational support. Peer health workers (also known as *Ssenga*, a respectful term meaning “aunty” in Luganda) were sex worker peers who openly declared the use of PrEP and had received training on PrEP use. Nakidde (highway community) explained that her *Ssenga* sent her text messages “asking why you did not get your medicine?” or to remind “us of the refill dates, time and where to go”. Peer health workers distributed PrEP from stock they held at their homes and provided dispensing records which were given to the health clinics each week. Nakidde (highway community) said, “for my refills, I just walk to [peer leader's] place and pick my medicine for about a month”. She also explained that if she was moving to different places of work for longer periods of time, then she asks her peer health worker “to give me PrEP for 2 months”. Diana (fishing community) concurred, saying “we tell *Ssenga* to bring us PrEP wherever we are”.

## Discussion

Drawing on Narasimhan et al's^3^ self-care framework which offers both people-centred and health system-centred perspectives on self-care, this qualitative study illustrates how self-care strategies enhanced the ability of female sex workers in rural Uganda to adhere to PrEP in their working and personal lives. While complementing existing research about PrEP use with female sex workers in urban settings in Uganda^9–11^ and sub-Saharan Africa,^12–14^ our research contributes specifically to a dearth of literature about how female sex workers living in *rural* Ugandan settings adhere to PrEP in their everyday lives. These findings also contribute to emerging literature^1–5^ that conceptualises how self-care can enhance the sexual health and well-being of marginalised and excluded people.

With regard to a people-centred perspective on self-care,^3^ our analyses explore how self-care is practised individually and evolves from, and is practised with, the support of others in their social and work settings. On an individual basis, women in our study described self-care strategies that integrated PrEP into their personal routines, at home and at work. Examples included reminders (i.e. setting an alarm on their phone to take tablets at the same time each day) and pairing PrEP with daily habits (i.e. keeping tablets near their cosmetics, where they pray each morning, next to a glass of water, or with their work equipment). On an interpersonal basis, our analyses illustrate how self-care strategies are embedded within women’s social lives. Women worked with support from a wide range of other people – long-term sexual partners, friends and neighbours – to maximise their capabilities to sustain PrEP adherence. A wide range of interpersonal self-care strategies evolved within these networks: verbal reminders to take tablets; information sharing; shared visits to attend clinics to collect PrEP refills and shared pill-taking routines with others with lived experience of HIV prevention or treatment. Our findings point to the diversity of ways in which female sex workers in this study accessed and used individual, familial and societal resources in their daily lives to build their agency to look after themselves – and others – in ways that reduced the risk of HIV acquisition in their working and personal lives. These shed light on how self-care strategies are overwhelmingly situated within relational and social contexts.^3^

A health systems-centred perspective on self-care^3^ illustrates the importance of support from health systems and health professionals for self-care, especially for self-management and self-awareness of health conditions.^1^ In our study, women described support in the form of information provision about all aspects of PrEP and organisation of PrEP refills through the use of card reminders. They perceived PrEP clinics managed by NGOs as friendly, safe, non-judgemental spaces in which they could talk freely about PrEP. Women described how health workers were perceived to go above and beyond normal working responsibilities – through the distribution of PrEP through home-based care outreach strategies, as well as in-bulk for periods of time women would be travelling to work. Such support for HIV self-care was complemented by the formal integration of female sex workers into the system of HIV care through training to become trusted peer health workers based on their existing lived experience of PrEP use. Our findings report the importance of health system and health professional support for people who often experience stigma and discrimination in society and health institutions due to socio-cultural and political stigma associated with their work. The safe linkage between self-care and quality healthcare for vulnerable individuals is critically important to avoid harm,^3^ and in this study was enabled by the flexibility of service delivery models within health systems.

More broadly, our research contributes to understandings of PrEP use among key populations in Uganda by emphasising distinct self-care strategies female sex workers in rural fishing and highway communities employ to support adherence. While previous studies in urban or peri-urban settings in East and Southern Africa have examined barriers to PrEP uptake and adherence,^9–14^ our results illustrate how women in rural areas develop innovative routine-based, and socially supported self-care strategies to incorporate PrEP into their daily lives, despite facing structural challenges and criminalised work environments. While studies in Kenya,^26^ South Africa^27^ and Zimbabwe^28^ highlight the importance of community-based support models, few studies have investigated what female sex workers living in rural areas do to integrate PrEP adherence into their daily lives. By doing this, and centring our analysis in rural Uganda where challenges of accessing fragmented health systems exist, our study contributes to a limited body of evidence on how people-centred approaches to supporting long-term PrEP use can be centred in women’s everyday lives.

### Study limitations

When interpreting our results, it should be noted that this was an exploratory qualitative study comprising a small sample size, due to restrictions related to COVID-19 and associated lockdowns that made recruitment difficult. Furthermore, women were recruited from well-organised clinic settings managed by NGOs that were firmly established in the area. It is possible that female sex workers who are less well connected to clinics and established outreach networks are underrepresented in this sample; this is a topic of possible future study. Thus care must be taken not to assume that this represents all female sex workers’ experiences of PrEP within or beyond these settings. “Internal reliability”^19^ is always a concern in rigorous qualitative research. This was enhanced in several ways. While not identifying as female sex workers, the Ugandan interviewers are well trained, with wide-ranging experiences of undertaking in-depth research with hard-to-engage populations about sensitive research topics, which enhanced deep engagement in the life worlds of study participants. Data was also collected in local languages and during sustained periods of immersive fieldwork in each setting, which enhanced local understanding of issues and built trusted relationships with young study participants. Furthermore, interviewers worked together with study investigators throughout the study to triangulate and discuss findings as data was collected, and to ensure rigour and consistency in data collection and interpretation.

### Implications for policy and practice

Despite these limitations, our analyses shed light on some activities and initiatives that can be implemented more systematically by the government and NGOs in Uganda – and in settings beyond Uganda – to support HIV prevention by enabling self-care among marginalised people, in spaces they deem safe to use. First, supporting female sex workers to identify and negotiate the existence of close, trusted and supportive networks – with sexual partners, peers, friends or neighbours – within which safe disclosure around the use of medication is possible can enhance and enable PrEP adherence. Second, self-care is complementary to, but not a replacement for, sustainable, high-quality HIV service and care provision.^5^ From this study, strategies that formalise peer-based support for self-care could support more widespread understanding of PrEP and enhance sustained attendance at refill meetings. Along with a community-driven model of care, other actions taken at the level of health services – such as PrEP refill cards, training health workers to know how to build trusting relationships and be responsive to sex workers’ needs, and models of home-based PrEP delivery – could become system-level norms of best practice to centre the needs of marginalised populations. Finally, though sex work is criminalised in Uganda, the health care system can safeguard the sexual rights of sex workers by enabling models of care that support inclusive, non-judgmental services that support female sex workers’ autonomy, choices and well-being.^1,3–5^ A self-care lens enriches understandings by emphasising the agency of female sex workers in efforts to enable PrEP adherence, even in settings of high vulnerability and limited structural support.

## Conclusion

PrEP is often perceived as a biomedical solution to HIV prevention in guidelines published by international agencies^7^ and national governments.^8^ Yet our findings point to the work that has to be done by people living with HIV, their families and communities, health professionals and health services, for PrEP to become effective. Drawing on recent people-centred and health system-centred conceptualisations of “self-care”,^3^ we have illustrated how PrEP adherence can be thought of as a set of self-care strategies, embedded within female sex workers’ personal and working lives, and supported by people in familial and social networks. These self-care strategies are enabled by health services and health professionals, and with careful support from health systems, can enable women to look after their health and well-being in ways that work best for them. A people-centred perspective on self-care illustrated a range of self-care strategies to support PrEP adherence conducted by women individually (medication reminders; pairing PrEP with daily habits), and with support from others in familial and social networks (verbal reminders to take tablets; information sharing; shared clinic visits; shared pill-taking routines). A health system-centred perspective illustrated the importance of support from health services and professionals, including through information provision; NGO clinics as friendly, safe, non-judgemental spaces; PrEP distribution through home-based care outreach strategies; in-bulk PrEP provision for work-related travel periods; formal integration of female sex workers into the system as peer health workers. By bringing person-centred and health system-centred perspectives on self-care together,^3^ we are able to ensure that health systems both recognise, and are designed to support, female sex workers and their communities as active agents in preventing HIV acquisition and transmission.

## Author contributions

*LJN, BB and AG designed the study. LJN and RN conducted the interviews, transcribed the interviews and performed initial data analysis while DS supervised the data collection and provided scientific oversight during study implementation. SB performed further analysis and commented critically on the manuscript. All authors read, edited and approved the final manuscript*.
